# Preoperative Visual Attention, Postoperative Delirium and Three‐Month Outcomes

**DOI:** 10.1002/alz70858_106799

**Published:** 2025-12-26

**Authors:** Benjamin A Chapin, Kathryn Pendleton, Faith Kimmet, Cynthia Garvan, Steven T. DeKosky, John B. Williamson, Catherine C. Price

**Affiliations:** ^1^ Malcom Randall VA, Gainesville, FL, USA; ^2^ University of Florida, Gainesville, FL, USA; ^3^ UF, Gainesville, FL, USA; ^4^ McKnight Brain Institute, Gainesville, FL, USA; ^5^ College of Medicine, University of Florida, Gainesville, FL, USA

## Abstract

**Background:**

Preexisting cognitive impairment is a significant risk factor for post operative delirium (POD), and POD increases morbidity and mortality. Disturbances of attention and awareness are necessary for delirium diagnosis, suggesting dysfunction in frontoparietal networks, which control visuospatial attention. However, preoperative visual attention has not been systematically investigated as a risk factor for delirium and adverse longitudinal outcomes in at‐risk adults. We provide an update from the study “Visual Attention and Postoperative Delirium” (AACSF‐22‐928731).

**Methods:**

In this prospective observational study, participants aged 65 years and older undergoing elective orthopedic surgery completed preoperative visuospatial attention measures, including the Posner cueing task. Our primary outcome was maximum POD severity, measured by the Confusion Assessment Method Severity (CAM‐S) short form. Secondary outcomes included falls, hospital readmission, and Zarit caregiver burden rating, measured three‐months post‐surgery. Data were summarized (i.e., frequencies, mean (SD), and median [Q1, Q3]) and analyzed (i.e., Spearman correlational testing).

**Results:**

The majority of the 45 participants were female (58%) and white (93%), with mean (SD) age 74.2 (5.4) years and 15.6 (2.7) years of education. 69% were not delirious post‐surgery, 18% were classified as subsyndromal (CAM‐S > 1), and 13% had POD. The delirium score's median [Q1, Q3] value was 0 [0, 2.0]. The median [Q1, Q3] value for stimulus reaction time was 510 ms [463,558]. The correlation coefficient (*p*‐value) between delirium and Posner stimulus reaction time was 0.45 (*p* < 0.001). Correlation coefficients were in the expected direction but not significant between Posner stimulus reaction time and three‐month outcomes. Delirium severity was correlated with caregiver burden (0.34, *p* = 0.044).

**Conclusion:**

Data continues to demonstrate that preoperative measures of visual attention predict POD. Additionally, POD measured in this study predicts caregiver burden at three months, supporting the importance of this finding. Further studies with more participants can help to determine how to apply measures of visual attention to better predict and monitor the effects of delirium on cognition and neurodegeneration. Understanding this relationship provides a basis for future research on strategies to prevent, mitigate, or rehabilitate the effects of delirium, improving hospital outcomes and potentially delaying or preventing dementia. Funding AACSF‐22‐928731; K07AG066813

